# Evaluation of Root Canal Morphology of Mandibular First and Second Premolars Using Cone Beam Computed Tomography in a Defined Group of Dental Patients in Iran

**DOI:** 10.1155/2017/1504341

**Published:** 2017-11-16

**Authors:** Neda Hajihassani, Neda Roohi, Karim Madadi, Mahin Bakhshi, Maryam Tofangchiha

**Affiliations:** ^1^Department of Endodontics, Dental School, Qazvin University of Medical Sciences, Qazvin, Iran; ^2^Private Practice, First Floor, No. 1, 7th Ave, Shohada Blvd, P.O. Box 3175745116, Fardis, Kara, Alborz Province, Iran; ^3^Department of Oral Medicine, Dental School, Shahid Beheshti University of Medical Sciences, Tehran, Iran; ^4^Department of Oral and Maxillofacial Radiology, Dental School, Qazvin University of Medical Sciences, Qazvin, Iran

## Abstract

**Background:**

Successful dental root canal treatments require a complete knowledge of dental anatomy and root canal morphology.

**Materials and Methods:**

One hundred and forty-five cone beam computed tomography (CBCT) images were used to assess the anatomy and morphology of mandibular premolars based on Vertucci's classifications in a defined group of dental patients in Iran. The number of roots and root canals, root canal morphology, root and canal shape (curvature), existence of C-shaped canal, and influence of sex on each of these were evaluated. A chi-squared test was used for statistical analysis.

**Results:**

The mandibular first and second premolars had a single root in 95.97% and 100% cases, respectively. In the mandibular first premolars, 62.2% were of type I, 0.8% type II, 10.9% type III, 0.8% type IV, 20.3% type V, 4.2% type VI, and 0.8% type VII; in the second premolars, 78% of canals were of type I, 3% type II, 11% type III, 7% type V, and 1% type VI. C-shaped canals did not exist in either of the premolars. The most prevalent root and canal shape was straight. The most prevalent root curvature was a distal curvature in both premolars (71.4% and 74% of first and second premolars, resp.). The most prevalent canal curvature was lingual and buccal for the first premolars (7.6% each) and distal for the second premolars (11%). No significant difference was found between men and women in nearly all of the above (*P* > 0.05).

**Conclusion:**

The results suggest that there is a need to conduct further evaluations on finding root and canal variations among more populations to gain better knowledge prior to root canal treatment.

## 1. Introduction

Nowadays, with the evolution of contemporary dentistry, endodontic treatment has become an important and effective method for maintaining and preserving dental health. Lack of knowledge of the internal anatomy of teeth and dental pulp is the second-most-important cause of treatment failures after wrong diagnosis and treatment planning [1]. Thus, an understanding of the internal anatomy of teeth is essential for better debridement and obturation of canals and, therefore, a better prognosis of the treatment.

The root canal system has a complex anatomy. It varies not only between different teeth but also in one particular tooth between different persons. Root canal anatomy does not usually reveal a single uniform tapered canal; extra canals, anastomosis, and other irregularities exist [2].

Mandibular premolars have one of the most complex anatomies. Treatment failures and posttreatment flare-ups have been reported in these teeth, thus indicating the existence of too much variation in their morphology [1, 3]. There have been several studies evaluating the morphology of mandibular premolars' root canal systems [4–8].

Several tools have been used to help recognize the anatomy of the root canal system [1, 9]. The identification of pulpal anatomy may be achieved through theoretical information from textbooks, radiographic evidence, and exploration of the canals during access cavity preparation [2].

Common intraoral and panoramic radiographs only provide a two-dimensional image and miss the important third dimension, which could lead to missing canals and the buccolingual curves of root canals. Cone beam computed tomography (CBCT), since its introduction in 1990, provides three-dimensional high-resolution images with the possibility of removing the superimposed structures; it could help in a better determination of root canal anatomy and morphology in a noninvasive way [10–13].

CBCT provides images in the axial, sagittal, and cross-sectional planes, as well as images of panoramic and three-dimensional reconstructions [13, 14]. In addition, it has a significantly lowered radiation dose compared to medical computed tomography (CT) [15–17].

Neelakantan et al. showed that CBCT offers accuracy as well as direct results (modified canal staining and clearing techniques) in the identification of root canal anatomy [18].

Michetti et al. compared two-dimensional coronal reconstructions of CBCT images with histological sections in the identification of root canal morphology and reported a strong to very strong correlation between CBCT-acquired data and experimental findings [12].

CBCT images can enhance the understanding of the root canal system and its variations among different populations. Thus, we decided to perform a study on evaluating the root canal morphology of mandibular premolars in an Iranian population, using CBCT images.

## 2. Materials and Methods

One hundred and forty-five CBCT images, including those of mandibular premolars and matching the following criteria, were selected: (1) teeth without periapical lesions; (2) non-endodontically treated teeth; (3) no open apex canals; and (4) CBCT images of high quality. The CBCT images were acquired using a Promax 3D CBCT unit (Planmeca Oy, Helsinki, Finland) at 84 kVp and 10 mA with an exposure time of 12 s. The voxel size of the images was 0.3 mm.

All the images were observed with Romexis Viewer 3.8.3.R. One radiologist and two endodontists evaluated the images at the same time until they reached a consensus and the final result was recorded in prepared forms. The number of roots and root canals, root canal morphology, root and canal shape (curvature), existence of C-shaped canal, and the influence of sex on each of these were evaluated. Vertucci's classification [19] was used to determine the type of root canal morphology. Vertucci's classification is defined as follows: type I (1), type II (2-1), type III (1-2-1), type IV (2), type V (1-2), type VI (2-1-2), type VII (1-2-1-2), and type VIII (3).

*Statistical Analysis*. SPSS 20 software and descriptive analytic tests were used for statistical analysis. Chi-squared and ANOVA tests were used to compare qualitative variants.

## 3. Results

In this study the CBCT archive of a maxillofacial radiology clinic in Qazvin, Iran, was evaluated. 224 out of total 985 CBCT records met the inclusion criteria.

One hundred and twenty-four first premolars and 100 second premolars were evaluated, of which 55 first premolars (44%) belonged to men and 69 (55.6%) to women, while 43 second premolars (43%) belonged to men and 57 (57%) to women. The average age was 40.4 years (21–66 years).

### 3.1. Mandibular First Premolars

As many as 119 (95.9%) had one root and five (4.1%) had two roots. In men, fifty-two (94.5%) had one root and three (5.5%) had two roots; meanwhile, 67 of women (97.1%) had one root and two (2.9%) had two roots. There was no significant difference between men and women in terms of the number of roots of the first premolar (*P* > 0.05).

The most prevalent canal configuration was type I (62.2%) in both men and women, which was more in women (70.2%) than men (51.9%). The second-most-prevalent type was type V (20.3%) in both genders, which was more in men (28.8%) than in women (13.5%). However, these differences were not significant (*P* = 0.067,* X*^2^ = 8.75) (Table 1).

Three out of five first premolars with two roots had two type I root canals in both roots. The other two had one root that branched into two roots at the apical third of the root and so did their root canals.

**Figure 1:**
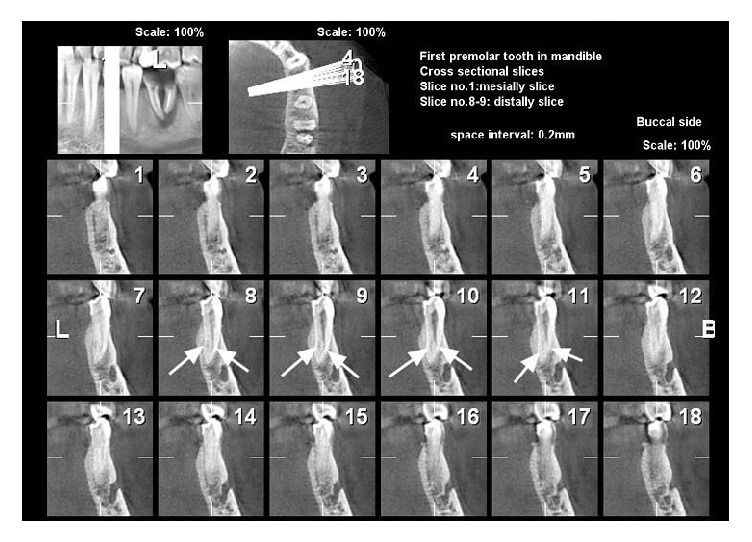
Mandibular first premolar with 2 canals in cross-sectional view (Vertucci's classification type III).

**Figure 2:**
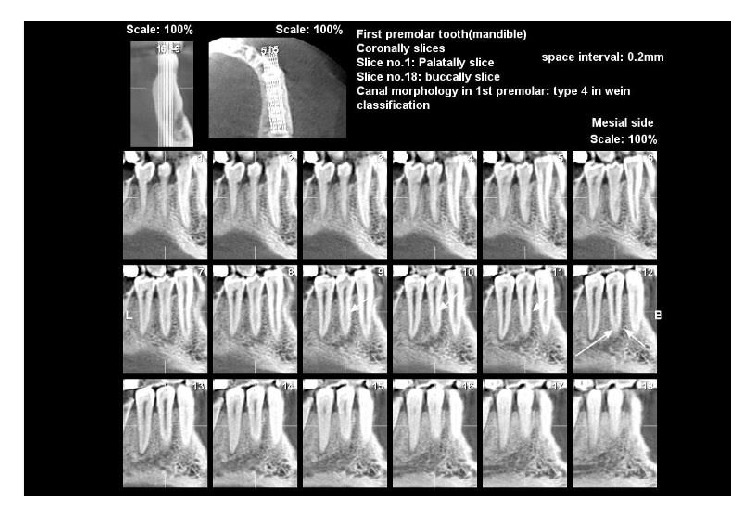
Mandibular first premolar with 2 canals in coronal view (Vertucci's classification type III).

The morphology of root canals using CBCT is shown in Figures 1 and 2.

The most prevalent root shape (curvature) was straight (71.4%); the distal curve at the second level was much less common (10.1%). The least prevalent curve was the buccal curve (0.8%). There was no significant difference between men and women (*P* = 0.158,* X*^2^ = 5.19) (Table 2).

The most prevalent canal shape (curvature) was straight (78.2%), and the buccal and lingual curves at the next levels were far less common (each 7.6%). The mesiobuccal curve was the least prevalent type, accounting for 0.8% of cases. Again, there was no significant difference between men and women (*P* = 0.161,* X*^2^ = 5.153) (Table 3).

Four out of five first premolars with two roots had no apical curve and; in one case, the roots had a distal curve.

### 3.2. Mandibular Second Premolars

The most prevalent root canal configuration was type I (78%) and the second-most-prevalent type was type III (11%) and type V (7%) in both men and women. There was no significant difference between the two genders (*P* = 0.152,* X*^2^ = 3.77) (Table 4).

The most prevalent root shape (curvature) was straight (74%), which was more in women (78.9%) than men (67.4%). This difference was significant (*P* = 0.08,* X*^2^ = 16.69). The distal curve was the second-most-prevalent curvature (10%). The mesial and buccal curves were the least prevalent (each with 2%) (Table 5).

The most prevalent canal shape (curvature) was straight (70%), followed by the distal curve (11%). There were no significant differences between the genders (*P* = 0.69,* X*^2^ = 1.45) (Table 6).

## 4. Discussion

Proper knowledge of root canal anatomy and morphology, as well as of anatomical variations between different teeth and in one tooth among different people, is an essential prerequisite for successful root canal therapy; a failure in this part could lead to a failure of the treatment [20–22]. Genetic and racial variations are factors that may affect root canal anatomy and morphology. Since mandibular premolars have complex anatomies, studies regarding anatomical variations in these teeth could lead to better knowledge prior to root canal treatment. Specific types of canal morphology occur in different racial groups [2]. For example, African-Americans have a higher number of mandibular premolars with extra canals. These patients had more than one canal in 33% of the first premolars and 8% of the second premolars; this was in contrast to Caucasians, who had multiple canals in 14% of the first premolars and 3% of the second premolars [2, 8]. Cleghorn et al. stated that higher incidences of teeth with additional canals and roots have been reported in Chinese, Australian, and sub-Saharan African populations, and the lowest incidence was in Western Eurasian, Japanese, and American Arctic populations [5].

There are several tools for the evaluation of root canal anatomy. Using CBCT images has many advantages, such as having results comparable to those of direct methods [12, 18]. Furthermore, a large area is imaged in one CBCT radiograph, and the adjacent and sometimes even cross-side teeth may be recognized. This may help in an exact determination of the tooth type in some cases. Many studies have evaluated the anatomy and morphology of the root canal system using CBCT images [10, 12, 18, 23–33] and some of them have studied mandibular premolars [10, 26–29, 33].

In our study, 95.9% of the mandibular first premolars and 100% of the second premolars had one single root. Yu et al. and Mirzaei et al. also reported the presence of more than one root more in the first premolars than in the second premolars [10, 33]. Although the difference between the two premolars was not significant in any of the studies, it should be considered. Shetty et al., while studying a south Indian population, reported all 126 mandibular first and second premolars to have a single root [29]. Cleghorn et al., in a literature review, reported 98% of the first premolars as single-rooted, 1.8% as having two roots, 0.2% with three roots, and the rare presence of four roots in less than 0.1% of cases [5]. However, in this study, only 4.1% of the first premolars had two roots and more roots were not present. Also, no significant difference was found between men and women in terms of the number of roots, which was consistent with other studies in this field [10, 26–29, 33].

In the present study, the most common canal configuration was type I Vertucci in both the first and second premolars; 62.2% of the first and 78% of the second premolars showed this canal type. Type V with 20.3% and type III with 10.9% were the next-most-common canal configurations in the first premolar. However, type III with 11% was the second-most-prevalent type among the second premolars and only 7% showed type V Vertucci. Other studies have reported type I as the most prevalent canal configuration in both premolars [10, 26–29, 33]. However, the second-most-prevalent canal type of the first premolar was type V, according to our study (20.3%), as well as according to the findings of Yu et al. (9.8%), Mirzaei et al. (13.6%), Liao et al. (8.8%), and Shetty et al. (12.3%). The second-most prevalent canal configuration in the second premolar was type III in our study (11%) and type V in the studies of Shetty et al. (7.5%) and Yu et al. (1.7%). In general, the mandibular first premolar has been reported to have more than one canal rather than the second premolar [10, 26, 27, 33]. In other words, the first premolars have a significantly greater variability in root canal morphology than the second premolars.

The most prevalent root canal configuration was type 1 Vertucci as revealed in a study on 2912 mandibular first and second premolars from several cities in Iran in 2016 [34].

Contrary to our results, another study in Kerman reported type 3 root canal configuration as the most frequent one. The difference in results might be attributed to insufficient sample size in the latter study [35].

Shahi et al.'s [6] and Khedmat et al.'s [36] studies were the most comparable ones to our study in regard to root canal configuration types [6, 36].

Our results regarding the mandibular 2nd premolar were different from other studies in Iran. The reported frequency of type 1 in Iran and other countries seems similar; however other types of root canal configuration showed different frequencies among countries. The difference might be mostly due to diagnostic techniques used in the studies such as CBCT, spiral CT, clearing, Indian ink, and methylene blue. In addition, several factors like ethnicity, gender, and geographic distribution can affect the root canal configuration [34].

No C-shaped canal was observed in our study, whereas some of the studies have reported the presence of C-shaped canals in the mandibular premolars [10, 28, 29, 33].

In our study, the root and canal curvatures were evaluated as well. The most prevalent root shape was straight for both premolars and the most prevalent root curvature was distal for both. The most prevalent canal shape was straight for both premolars as well. The most prevalent curvature was the lingual and buccal curvature for the first premolar and the distal curvature for the second premolar.

There were no significant differences between men and women in any of the above, except for women having a significantly higher incidence of the straight root shape than men.

## 5. Conclusion

Variability is observed between different studies conducted in different populations regarding the root canal configuration of mandibular premolars. However, all the reviewed studies pointed out that most of the mandibular premolars have a single root. The most prevalent root canal type at both premolars was type I Vertucci. Moreover, most of the mandibular premolars have straight roots and canals, and there was no significant difference between men and women in nearly all of the above.

## Figures and Tables

**Table 1:** Root canal morphology of mandibular first premolars based on Vertucci classification.

Canal type	Women	Men	Total
	count (%)	count (%)	
I	47 (70.1)	27 (51.9)	74 (62.2)
II	1 (1.5)	0 (0.0)	1 (0.8)
II	6 (9.0)	7 (13.5)	13 (10.9)
IV	0 (0.0)	1 (1.9)	1 (0.8)
V	9 (13.4)	15 (28.8)	24 (20.2)
VI	4 (6.0)	1 (1.9)	5 (4.2)
VII	0 (0.0)	1 (1.9)	1 (0.8)
VIII	0 (0.0)	0 (0.0)	0

*Total*	*67*	*52*	*119*

**Table 2:** Root shape (curvature) of mandibular first premolars.

Root shape	Women	Men	Total
	count (%)	count (%)	
Straight	50 (74.6)	35 (67.3)	85 (71.4)
Distal curve	7 (10.4)	5 (9.6)	12 (10.1)
Distolingual curve	2 (3.0)	5 (9.6)	7 (5.9)
Lingual curve	2 (3.0)	4 (7.7)	6 (5.0)
Mesial curve	4 (6.0)	0 (0.0)	4 (3.4)
Distobuccal curve	1 (1.5)	1 (1.9)	2 (1.7)
Mesiobuccal curve	1 (1.5)	1 (1.9)	2 (1.7)
Buccal curve	0 (0.0)	1 (1.9)	1 (0.8)

*Total*	*67*	*52*	*119*

**Table 3:** Canal shape (curvature) of mandibular first premolars.

Canal Shape	Women	Men	Total
	count (%)	count (%)	
Straight	55 (82.1)	38 (73.1)	93 (78.2)
*Lingual* curve	2 (2.9)	7 (13.5)	9 (7.6)
*Buccal* curve	4 (5.9)	5 (9.6)	9 (7.6)
*Distal* curve	3 (4.5)	1 (1.9)	4 (3.4)
*Distolingual* curve	2 (2.9)	1 (1.9)	3 (2.5)
*Mesiobuccal* curve	1 (1.5)	0 (0)	1 (0.8)

*Total*	*67*	*52*	*119*

**Table 4:** Root canal morphology of mandibular second premolars based on Vertucci classification.

Canal type	Women	Men	Total
	count (%)	count (%)	
I	46 (80.7)	32 (74.3)	78
II	1 (1.8)	2 (4.7)	3
II	4 (7)	7 (16.3)	11
IV	0 (0)	0 (0)	0
V	5 (8.8)	2 (4.7)	7
VI	1 (1.8)	0 (0)	1
VII	0 (0)	0 (0)	0
VIII	0 (0)	0 (0)	0

*Total*	*57*	*43*	*100*

**Table 5:** Root shape (curvature) of mandibular second premolars.

Root Shape	Women	Men	Total
	count (%)	count (%)	
Straight	45 (78.9)	29 (6.4)	74
Distal curve	6 (10.7)	4 (9.3)	10
Distolingual curve	2 (3.6)	3 (6.9)	5
Lingual curve	1 (1.7)	3 (6.9)	4
Distobuccal curve	1 (1.7)	2 (4.7)	3
Buccal curve	1 (1.7)	1 (2.3)	2
Mesial curve	1 (1.7)	1 (2.3)	2

*Total*	*57*	*43*	*100*

**Table 6:** Canal shape (curvature) of mandibular second premolars.

Canal Shape	Women	Men	Total
	count (%)	count (%)	
Straight	39 (68.4)	31 (72.1)	70
Distal curve	8 (14.1)	3 (7.0)	11
Lingual curve	5 (8.7)	4 (9.3)	9
Buccal curve	3 (5.3)	4 (9.3)	7
Distolingual curve	2 (3.5)	0 (0)	2
Mesial curve	0 (0)	1 (2.3)	1

*Total*	*57*	*43*	*100*
